# Working practices and incomes of health workers: evidence from an evaluation of a delivery fee exemption scheme in Ghana

**DOI:** 10.1186/1478-4491-5-2

**Published:** 2007-01-22

**Authors:** Sophie Witter, Anthony Kusi, Moses Aikins

**Affiliations:** 1 Immpact, University of Aberdeen, Health Sciences Building, Foresterhill, Aberdeen AB25 2ZD, Scotland, UK; 2 Immpact, Noguchi Memorial Institute for Medical Research, University of Ghana, Legon, Accra, Ghana

## Abstract

**Background:**

This article describes a survey of health workers and traditional birth attendants (TBAs) which was carried out in 2005 in two regions of Ghana. The objective of the survey was to ascertain the impact of the introduction of a delivery fee exemption scheme on both health workers and those providers who were excluded from the scheme (TBAs). This formed part of an overall evaluation of the delivery fee exemption scheme. The results shed light not only on the scheme itself but also on the general productivity of a range of health workers in Ghana.

**Methods:**

A structured questionnaire was developed, covering individual and household characteristics, working hours and practices, sources of income, and views of the exemptions scheme and general motivation. After field testing, this was administered to 374 respondents in 12 districts of Central and Volta regions. The respondents included doctors, medical assistants (MAs), public and private midwives, nurses, community health nurses (CHNs), and traditional birth attendants, both trained and untrained.

**Results:**

Health workers were well informed about the delivery fee exemptions scheme and their responses on its impact suggest a realistic view that it was a good scheme, but one that faces serious challenges regarding financial sustainability. Concerning its impact on their morale and working conditions, the responses were broadly neutral. Most public sector workers have seen an increased workload, but counterbalanced by increased pay. TBAs have suffered, in terms of client numbers and income, while the picture for private midwives is mixed. The survey also sheds light on pay and productivity. The respondents report long working hours, with a mean of 54 hours per week for community nurses and up to 129 hours per week for MAs. Weekly reported client loads in the public sector range from a mean of 86 for nurses to 269 for doctors. Over the past two years, reported working hours have been increasing, but so have pay and allowances (for doctors, allowances now make up 66% of their total pay). The lowest paid public health worker now earns almost ten times the average gross national income (GNI) per capita, while the doctors earn 38.5 times GNI per capita. This compares well with average government pay of four times GNI per capita. Comparing pay with outputs, the relatively high number of clients reported by doctors reduces their pay differential, so that the cost per client – $1.09 – is similar to a nurse's (and lower than a private midwife's).

**Conclusion:**

These findings show that a scheme which increases demand for public health services while also sustaining health worker income and morale, is workable, if well managed, even within the relatively constrained human resources environment of countries like Ghana. This may be linked to the fact that internal comparisons reveal Ghana's health workers to be well paid from public sector sources.

## Background

The Millenium Development Goals set a target of reducing maternal mortality by three-quarters by 2015 [1]. So far, relatively little progress has been made, and donors and governments are looking for cost-effective and sustainable approaches which can reduce persistently high maternal mortality. One of the main challenges encountered has been to increase the proportion of women receiving antenatal care and delivering with a skilled attendant. Globally, only 43% of women receive skilled care at their deliveries [2]. Increasing this is a key component of the Safe Motherhood Initiative [3].

Studies have found a number of barriers to women delivering in health facilities [4,5]. These include cultural preferences, distance to facilities, poor quality services, and negative attitudes of health workers. Financial barriers have also been noted as an important issue, but there have been few studies to investigate whether delivery fees influences women's uptake of maternal health services.

Institutional data indicate that Ghana has persistent high maternal mortality ratios, estimated to range from 214 to 800 per 100 000 live births [6]. It also has growing social inequalities, with rates of skilled attendance either static or declining for poorer women [7]. Nationally, 45% of births are attended by a skilled attendant (defined here as a doctor, MA, nurse or midwife), 30% by traditional birth attendants (TBAs), and 25% are unsupervised. There is a significant rural/urban difference (29% in urban areas; 33% in rural) as well as regional variations. The three northern regions have the highest levels of poverty and maternal mortality and the lowest levels of supervised deliveries [8].

While financial barriers are only one of the constraints to seeking skilled care during deliveries, they are believed to be one of the most important factors. A costing study found that clients paid an average of $12 for vaginal deliveries in public hospitals and $20 in mission hospitals, while caesareans cost an average of $68 in public hospitals and $139 in mission hospitals, with user fees constituting most of this total [9].

The Government of Ghana introduced a scheme to exempt users from delivery fees in September 2003 in the four most deprived regions of the country, and in April 2005 it was extended to the remaining six regions of the country. The aim of the delivery fee exemptions policy (DFEP) was to reduce the financial barriers to using maternity services. It was expected that this would lead to a reduction in maternal and perinatal mortality, as well as contributing to poverty reduction [10].

The DFEP scheme was funded through Highly Indebted Poor Country (HIPC) debt relief funds, which were channelled to the districts to reimburse facilities – both public and private – according to the number of deliveries which they performed each month. A tariff was approved by the Ministry of Health which set reimbursement rates according to the type of delivery (such as 'normal', 'assisted delivery', or 'Caesarean section') and the facility type (mission and private facilities are reimbursed at a higher rate, in recognition of the fact that they do not receive public subsidies) [10]. Women were supposed to face no direct costs as a result of their delivery, other than those incurred in reaching facilities.

In 2005, an evaluation of the DFEP started, focussing on how it has affected utilisation, quality of services and health and non-health outcomes for households [11]. This paper describes one component – a survey of health workers who are directly involved in deliveries. The aim was to establish the impact of the delivery fee exemptions scheme on health workers, their income, workload, working conditions and motivation. This was felt to be of particular importance as human resources are regarded as the most constraining factor in the health system in Ghana [12-14] due to the well-documented brain drain and also the difficulty of retaining health staff in rural areas.

Table 1 indicates the hypotheses behind this survey – the channels through which the policy might affect health workers and the expected direction of change of the different indicators. In particular, there was a concern that the exemptions scheme would increase the workload of existing overstretched staff, and that this would contribute to demoralisation and a consequent drop in quality of services for clients. A separate concern was that health workers' incomes might suffer. This paper investigates whether this concern was justified and what the findings show about the workload and productivity of health workers in Ghana in general.

**Table 1:** Research questions for health worker incentives survey, Ghana

Type of impact	Hypotheses about how the delivery fee exemption policy might affect HWs and TBAs	Indicators and expected direction of change
Changes to income	1. Main salary, allowances, per diems and benefits in kind not workload-related so should not change for public and mission staff. For private midwives, though, income is related to work, so will reflect demand for their services under the new policy.	Income from different sources collected to put other variable sources in perspective, but any change assumed to be unrelated to DFEP, except for private midwives.
	2. Private practice income might be reduced, if hours spent in public service increased, with increased demand.	Private practice hours, client numbers and income might decline, for public and mission HWs.
	3. Incentive income related to the DFEP might redress any losses from other categories.	DFEP incentive payments monitored, for all groups other than TBAs.
	4. Anecdotally, staff used to make pocket money from sales of items to women coming in for deliveries; this might be jeopardised by policy.	HWs asked about sales to patients. Reduction expected.
Changes to working hours and work load	5. Would expect policy to increase working hours and workload for all HWs, public and private, and to diminish them for TBAs, who excluded from the policy*.	Working hours for main job, number of clients seen, and number of deliveries performed are all expected to rise, except for TBAs, where expect a drop (at least relative drop, allowing for population growth).
Changes to general motivation	6. Might expect working conditions to improve, if funding for scheme is sustained and drugs and supplies are easily acquired; or the reverse, if funding is inadequate.	Unclear direction, but indicators are answers to questions on HW views of the policy's impact (particularly in relation to drugs and supplies).
	7. Psychological benefits from knowing that all clients can access services and are not struggling to pay their bills. Staff no longer have to 'help' financially challenged women	HW views on the policy – expect positive reports on the overall impact of the scheme.
	8. TBAs may be struggling and hostile to a policy which has negatively affected their business. Private midwives may also be disaffected if payments under the DFEP are less than they used to raise from user payments.	TBAs' and private midwives' views of the DFEP – expect negative reports from TBAs and ambivalent from private midwives.

* According to national guidelines, TBAs were not included. However, in one district in Volta, the district opted to include them in the DFEP, providing small payments per women delivered.

## Methods

For the evaluation, two regions were chosen; one which was part of the first group to join the DFEP (Central), and one from the second phase (Volta). Within each region, six districts were chosen, matched for characteristics such as population size, poverty status, urban profile and health infrastructure [11].

A structured questionnaire was developed during 2005, looking at individual and household characteristics, working hours and practices, sources of income, and views of the exemptions scheme and general motivation. Many of the questions probed for change over the previous two years, in order to assess changes which might be related to the introduction of the DFEP. Most of the questions were closed, though there were two open-ended questions at the end on the respondents' opinions of the exemption scheme and on their recommendations for its future. After field testing, this was administered to health workers and TBAs in November 2005. TBAs were not included in the DFEP (with the exception of one district), but were surveyed in order to see how their business had been affected by clients gaining free deliveries with health workers. A shortened version was administered to TBAs, translated into the local languages.

Sampling was based on estimated total numbers within various professional categories directly involved in deliveries. As some of these groups are small, the aim was to capture virtually all members of smaller groups (doctors); approximately 50% of midwives and nurses and 10% of TBAs. Within these groups, sampling was both random and convenient.

Approximately 75% of the desired sample interviews were achieved, partly due to inaccuracies in the sampling frames and partly due to the difficulty of locating some of the groups, particularly untrained TBAs.

Data was entered into EpiInfo, cleaned and then analysed using SPSS 14. Responses to open-ended questions were grouped thematically and then analysed in terms of their frequency.

## Results

### Respondent and household characteristics

In all, 374 health workers and TBAs were interviewed in the study (Table 2). Midwives formed the largest group, representing 36% of the respondents; this was followed by trained TBAs (29%). The other respondents consisted of doctors (6%), medical assistants (3%) nurses (13%) and untrained TBAs (13%).

**Table 2:** Survey sample, by professional title and region

***Professional title***	***Region***		***Total***
		
	***Central***	***Volta***	
Doctor	12	9	21
Medical assistant	3	8	11
Public midwife	59	58	117
Private midwife	12	4	16
Nurse	17	22	39
Community nurse	10	1	11
TBA (trained)	66	42	108
TBA (untrained)	32	19	51
Total	211	163	374

The majority of respondents were females with males constituting only 11%. The doctors were predominantly males (95%) while all the midwives were females. It is interesting to note that 6% of the trained TBAs and 14% of the untrained TBAs surveyed were males.

Most respondents were married with children (58%). The age range was 24 years to 90 years, with a mean of 53 years. The oldest professional group, on average, were the trained TBAs (63), followed by untrained TBAs (58) and private midwives (58).

The majority of TBAs had no formal education. The majority of the doctors (81%) had basic degrees while 19% had postgraduate degrees. For all other groups, a Certificate in Nursing or Midwifery was the most common qualification.

The most common place of work for TBAs was their home, though a small proportion was based in prayer camps. For doctors, midwives and nurses, the district hospital was the most common place of work, while the largest proportion of MAs were based in health centres and community health nurses (CHNs) in mission clinics. (Mission staff in Ghana are generally on secondment from public service and paid by the government, and are therefore considered as public sector workers for most purposes.)

Household size (defined as including all adults and children who had slept in the dwelling for at least 3 months out of the previous 12) varied according to professional group, with a doctor's household (mean 3.8 people) approximately half the size of a TBA's (mean 8 people), though the doctors had a higher number of external dependents.

The respondent's income was generally the only or main source of income for the households; however, for around a third of TBAs it formed a supplement to the household income.

Monthly household income and expenditure was significantly higher in Volta Region than in Central. Average household expenditure was ¢2.84 million in Volta, compared to in ¢1.85 million in Central; average household income was ¢3.21 million in Volta, compared to ¢2.55 million in Central. (The exchange rate during the survey year, 2005, averaged ¢9,000 per US dollar.)

Taking into account income, savings and borrowings, net household revenue ranged from ¢9.18 million for doctors to ¢415 270 for TBAs.

Of the whole interviewee group, 70% had electricity at home; 56% had a TV set; 76% had a radio; 35% had a video; 47% had a fridge; 36% had a telephone; 38% had running water; and 71% had a toilet or pit latrine. However, TBAs had significantly fewer of these assets than the health workers.

### Working practices and workload

The respondents were experienced health workers, evident by the high mean number of years in the profession, which varied from 12 years for CHNs to 30 years for MAs.

The reported mean number of hours worked per week varied greatly between professional groups. TBAs largely worked part-time, with a mean of 20 hours per week, whilst all other health workers completed significantly more hours, ranging from a mean of 56 hours per week for CHNs to 129 per week for MAs. 88% of respondents reported an increase in working hours over the past two years. The net change ranged from a decrease of 6 hours per month for private midwives to an increase of 23 hours per month for MAs.

99% of respondents reported no other employment. However, 45% of TBAs had other income generation (IG) activities, which they spent on average 4.4 hours on per week. Most had seen no change (85%), and change where it had occurred took the form of a small increase in time (2.5 hours extra per week on average).

All groups, apart from TBAs, reported a net increase in clients (ranging from 7–17 extra clients per week) over the past two years. There was no significant difference in client load between the regions, but Volta had seen a greater increase over the past two years.

The workload of the respondents was assessed by the mean client numbers per week. The mean clients seen per respondent in a week ranged from 109, among the doctors, to a low of 6, among the untrained TBAs. Public midwives attended to 119 clients on average, while the private midwife attended to an average of 38 clients.

**Figure 1:**
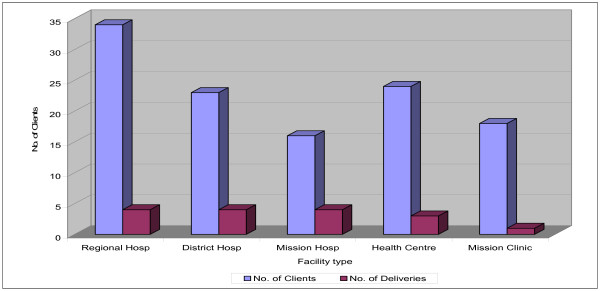
Mean daily workload per public midwife, by facility type.

The reported daily workload of a midwife at the regional hospital averaged 34 clients, compared with 24 at the health centre, and 23 at the district hospital, on an eight hour shift schedule (Figure 1).

Public midwives also reported carrying out the largest number of deliveries per week, with 19 on average, as against 4 by private midwives. The lowest number of deliveries per week was for untrained TBAs, who performed an average of 2 deliveries per week. The majority of respondents reported an increase in numbers of deliveries per week over the past two years, with public midwives most affected (a net increase of 5 deliveries per week on average). Only TBAs registered a decrease, of 1 delivery per week on average per TBA. Regional differences in delivery numbers were not significant.

In terms of place of delivery, district and regional hospitals were the busiest, with 18 deliveries per week on average. There was no significant difference in the mean daily number of deliveries per public midwife working in different facilities. The mean was four deliveries per day for those at the regional, district and mission hospitals, and three per day for those at the health centres.

### Personal income

Reported health worker salaries ranged from ¢868 000 per month for CHNs to ¢2796 million for doctors. Private midwives earned significantly more than public ones. Trained TBAs earned ¢106 000 on average, while untrained TBAs earned ¢78,000, but 25% reported that they earned no income from deliveries, either because they were assisting friends and family or because clients had failed to pay them.

70% of respondents reported a change in income over the two years preceding the study, with 88% reporting an increase in their monthly salaries. The largest increase among the public health sector workers was ¢636 722, reported by doctors.

In addition to salary, allowances are an important source of income for all health workers (TBAs were not asked about allowances as they do not receive them). 15% reported receiving Deprived Area Allowance (DAA), ranging from ¢1.275 million per month for doctors to ¢195 000 for CHNs. A higher proportion of DAA recipients were in Central Region, which has more districts classified as deprived. 75% of those receiving DAA described it as a fixed allowance.

Additional Duty Hours Allowance (ADHA) was received by all the public sector health workers interviewed, with the average ranging from ¢1.115 million per month for CHNs to ¢4.789 million for doctors. Although the ADHA was originally intended as a bonus for working extra hours, 97% of respondents described the ADHA as a fixed payment. Only 60% reported that it was paid on time.

Only 12% of health workers reported receiving an incentive payment related to deliveries. Almost all were in Central Region. These ranged from ¢70 000 per month for CHNs to ¢245 000 for midwives. 12 trained TBAs from Nkwanta district, Volta, and one untrained TBA from Central, also reported receiving delivery exemption incentives. Delivery exemption incentives were thought to be workload-related (84%).

The main reported increase in allowances was for ADHA, which had risen by an average of ¢520,000 per month for CHNs and ¢1.514 million for doctors.

Public sector health workers also received additional benefits as part of their jobs. Doctors received the greatest number of these benefits, which included accommodation (75%), food (25%), free health care (75%) and a car (10%). Housing and health care were the main benefits for other public sector health workers, though the numbers receiving them were much lower.

The proportion of health workers who received some share of the internally generated funds (IGF, or user fee) revenue coming into their facility was 14%, though these were concentrated in certain districts. These monies are not meant to be shared with staff. It may be that staff were reporting some sort of cash advance from facilities or ex gratia gifts for special occasions. Half of all doctors, a quarter of nurses and a small proportion of midwives reported receiving some payment from IGF. MAs and CHNs did not receive any. The payment to doctors ranged between ¢1 000 000 and ¢4 000 000 per month while that to nurses ranged between ¢20 000 and ¢50 000.

The proportion reporting income from per diems ranged from 36% for MAs to 80% for doctors; adding an average of ¢224 000 and ¢873 000 respectively to the monthly income of those receiving them.

Income from private practice was only reported by 1% of health workers.

Total income from all sources ranged from a mean of ¢78 000 per month for untrained TBAs to just under ¢11 million for doctors. Salaries formed only 34% of doctors' total pay.

### Motivation and views of exemption scheme

Respondents were asked to rank the factors that motivated them to stay in their jobs. The most popular response was opportunity to serve the community, with 66% selecting this option; next came the social status attached to the profession (8.3%); then opportunities for training (4.3%). Only 3.8% felt that allowances were the most important factor, while 1.6% put their salary as most important and 0.3% put good working conditions. Ironically, those who identified pay as important were the ones being paid the least – the TBAs.

95% of respondents were aware of the delivery fee exemptions scheme (those least aware were TBAs, who had not been included in the scheme). Most had been informed by official channels or circulars (for health workers) or by midwives (for TBAs). 87% reported that exemptions were in effect in their facilities at the time of the survey (November 2005).

When asked for their opinion of the scheme and its implementation, the main opinions expressed were that it had lessened the burden on the poor (24%); that it had increased facility-based deliveries (20%); and that, although the scheme was good, it was not sustainable (16%).

Respondents were asked to agree or disagree with statements about the effect of the scheme on service delivery. Responses from Central Region were generally more positive. Over 99% of respondents in both Central and Volta regions felt that more women were now delivering with skilled assistance and that the scheme had benefited the poor. About 76% in Central Region (CR) and 59% in Volta Region (VR) thought that there were enough drugs and supplies to deal with the increasing number of deliveries. Only 29% in Central and 19% in Volta regions thought that there was enough staff to handle the increasing numbers of deliveries.

Respondents were also asked about the effects of the scheme on them personally. Approximately 61% of all respondents felt that the scheme had increased their workload; however,46% of private midwives and more than 60% of TBAs felt that the scheme had eroded their client base. Dissatisfaction was highest among TBAs and private maternity home operators, who felt that their businesses had suffered as a result of the scheme. Many (40.8% in CR; 66.4% in VR) felt that the scheme had not changed their general working conditions.

When asked for their opinion of the scheme in an open-ended question, the main opinions expressed were that it had lessened the burden on the poor (24%); that it had increased facility-based deliveries (20%); and that it was good but not sustainable (16%). Concerns about sustainability were higher in Central, while concerns about the financial burden on facilities and the impact on staff workload were higher in Volta.

When asked for their recommendations in relation to this scheme, the most wide-spread request was for the regular release and payment of funds (24%); followed by improved working conditions for staff (18%); the provision of more logistics and supplies (14%); and training and recruitment of more staff (12%). In Central, the top three requests were for regular payments, more targeted coverage and the inclusion of TBAs in the scheme, while in Volta the emphasis was on improved working conditions for staff, training and recruitment, and logistics/supplies, as well as regular payments.

## Discussion

This survey relies on self-reporting by health workers, which clearly introduces a potential for bias. The likely direction of the bias in reporting would be upward for workload and working hours and downward for pay and other sources of revenue. We can therefore assume that the payments reported are conservative estimates.

Reported working hours are very high, especially for MAs, but these estimates may include on-call time as well as time which is actively worked. For workload, crude comparisons with facility reports on numbers of deliveries and numbers of staff working on deliveries [15] does suggest that the activities reported here are higher than expected, but we have to allow for the fact that each delivery will be attended by more than one member of staff. Increases in reported deliveries (which range from 11% to 36% for different groups of health workers) are higher than the overall reported increase in supervised deliveries (14% for Central Region for 2003–4, for example, and 6% for Volta for 2004–5, according to official RCH figures), but again, if staff are overlapping with one another, then these figures may still be consistent. Analysis of changes in supervised delivery during the implementation of the DFEP, based on an IMMPACT household survey, found a significant increase of 12% in Central Region, while the increase in Volta was not found to be significant [16].

A further caveat on accuracy of findings relates to the recall of health workers, given that many of the questions in the survey were seeking to identify changes over the two year period in which the policy had been introduced. Such recall is likely to be able to indicate the direction of change and its rough magnitude, rather than giving precise figures.

Questions on household income were difficult for respondents to answer and the answers given do not accord with responses to questions on personal income (for example, all of the groups apart from private midwives and TBAs report monthly household income levels below their personal monthly income, even before allowing for savings and borrowings). The analysis of findings has therefore focussed on personal income rather than household income.

Reported private practice was very low, which may be due in part to under-reporting, but was judged to be consistent with known patterns outside the Greater Accra region.

Questions on general motivation are indicative but clearly have to be interpreted with care. The high proportion of health workers reporting service to the community as their first motivation for doing their job did not fit well with the industrial action on pay and conditions which was launched shortly after this survey was administered (in spring 2006). While the debate continues on non-financial incentives versus financial incentives as motivators of health staff in developing countries, it is clear that financial incentives are important, even if they are not the only factors to consider [17].

Table 3 compares our expected changes with the actual results, to draw conclusions about the impact of the policy. As the policy change took place in a dynamic health sector context, these conclusions can only be tentative, but most of the indicators have changed in the direction predicted by our hypotheses. Workloads for public health workers have increased, but pay has also risen (as part of general public sector pay awards) to compensate. In Central Region, the incentive payments directly linked to the DFEP have provided motivation, especially for midwives. Qualitative responses indicate high awareness and an overall neutral impact for the public sector.

**Table 3:** Comparing results with predicted changes, health worker incentives survey, Ghana

**Indicators**	**Results**	**Comments**
1. Salary and allowances	Change in real terms of basic income (salary plus ADHA) over two years of: 30% for doctors and MAs; 37% for public midwives; 35% for private midwives; 28% for nurses; and 52% for community health nurses. TBAs seen drop in real income of 4% for trained and 16% for untrained.	General pay has risen for health workers. However, the fact that private midwifes have also increased their pay, while TBAs have seen a real decline, does suggest that the DFEP is affecting their incomes (positive for those groups included and negatively for the excluded groups).
2. Private practice	Private practice income was almost non-existent (only 1% – two doctors – reported any).	Given how small these elements are, no impact can be expected from the DFEP.
3. DFEP incentive payments	Exemption incentives were reported to have been received by only 11.6% of health workers. The public midwives reported the highest average payment of ¢245,000 per month. This incentive was mainly reported by respondents from the Central Region who constituted 93.5% of the recipients.	These incentives were not mandated nationally and therefore only affect regions which have decided to institute them. The figures for CR suggest that at nearly 19% of their basic salary, these incentives should have had a motivating effect on midwives.
4. Sales to patients	Virtually no reports of additional income for health workers – only 3 midwives reported any. Not much change reported either (one had increase slightly; others no change).	Very few responses, therefore no impact noted either way. However, there may be under-reporting (usual for informal payments etc.).
5. Working hours and client numbers	All groups record increase in working hours (21% for doctors; 22% for MAs; 27.5% for public midwives; 12% for nurses; 14% for CHNs), with exception of private midwives (who report a decrease of 5%) and TBAs (who report a decrease of 9% for trained and no change for untrained)	As population growth is in the region of 2.6% per annum, these large increases for public sector workers cannot simply be attributed to that. They suggest that the DFEP is increasing their workload. The picture is more complex for private midwives, who report and increase in clients and income, but a decrease in working hours and no change in deliveries. It may be that they are switching to other services or being paid more for the stable number of deliveries that they are performing. For TBAs the picture is clear: decline in income, working hours, clients and deliveries, which are likely to be attributable, at least in part, to the DFEP.
	All groups record increase in clients per week (7% for doctors; 11% for MAs; 17% for midwives; 36% for private midwives; 9% for nurses; 14% for CHNs), with exception of TBAs (who report a decrease of 11% for trained and 14% for untrained TBAs)	
	All groups record increase in deliveries per week (27% for doctors; 33% for MAs; 36% for midwives; 27% for nurses and 11% for CHNs), with exception of private midwives (no change) and TBAs (decrease of a third for both types).	
6. Working conditions	76% of respondents in CR and 59% in VR felt that drugs and supplies were adequate to deal with the increases in numbers. However, only 29% in CR and 19% in VR felt that staffing was adequate.	We do not know how satisfied staff were with supplies, staff numbers etc. before the policy – these factors were already constrained. However, it is clear that adequate staffing is their main concern in relation to the DFEP.
7. Psychological benefits to staff	Asked about the impact of the policy on them personally, 61% report an increase in workload; 61% report an increase in income; 42% report no change in job satisfaction; and the responses to overall change in work situation is fairly equally divided between improve, worsen and no change.	These responses reflect the changes reported above – more work, higher incomes and an overall neutral effect on general satisfaction.
8. TBAs' and private midwives' views	TBAs and private midwives were the only groups to report a decrease in their workload due to the DFEP. They and the mission sector report a decrease in income too, in open questions.	This correlates with findings above – negative impact on TBAs and ambivalent on private midwives.

TBAs, on the other hand, have been negatively affected in all districts except Nkwanta, where they were involved in the DFEP. For private midwives, the picture is mixed, with a reported increase in pay and client numbers but shorter working hours and fewer deliveries. It may be that in some areas private midwives are losing clients to better equipped (and now free) public facilities.

This survey also sheds light on the general working conditions for health workers and TBAs in Ghana. One striking feature is the extent to which public health workers focus on their main employment – their long hours and almost non-existent income from private practice testify to this. (Note that these results reflect the situation outside major urban centres. In Accra, the extent of private practice is likely to be much higher, for example.) This may, in part, be a result of the brain drain and staff retention problems, which have left facilities with staff shortages (vacancy rates of 47% for nurses and 57% for doctors in 2002 [13]).

The Government of Ghana has had to adopt a number of coping strategies to reduce the brain drain of doctors, nurses and midwives; including allowing staff to work to higher ages, relying more heavily on lower grade health workers, and increasing allowances. These strategies were evident in the results of this survey: the mean age of respondents was 53 years, which is relatively high; medical assistants reported working the longest hours of all the groups surveyed (129 hours per week) and were the main providers of services at grassroots health centre level; and allowances formed the majority of take-home pay for doctors, midwives and CHNs.

Set against the reported increase in workload over the past two years is the reported rise in incomes. This varies between professional groups, but public midwives, for example, report net increases of 35.7% in the number of deliveries performed, set against a net real increase in pay of 37.4% over the past two years and a net increase in working hours of 27.5%. This indicates that pay has more than kept up with increased output, and that productivity per hour worked has increased, at least for this group.

Although there are no set international norms for workloads, the mean client load reported in this study fits within the range of a study carried out in South Africa, which set norms at 20–35 patients per day per nurse (though the actual workload variation was found to be large: from 6 to 60 patients per day per nurse) [18]. This suggests that Ghana's health staff are not overloaded, even after the introduction of this policy which boosted demand for their services. Note however that client load is a crude indicator, which aggregates different types of encounters, some of which (like deliveries) are more time-consuming than others (like ante natal check ups).

How productive are these health workers? Table 4 gives some summary figures for pay and workload of the different groups surveyed in this study, and some crude productivity figures. It is hard to make international comparisons, but internal comparisons reveal that they are well paid. The lowest paid public health worker earns almost ten times the average GNI per capita, while the doctors earn 38.5 times GNI per capita. This compares well with an average government pay of four times GNI per capita. Compared with international rates for doctors, however, there will still be a 'pull' factor of higher pay elsewhere, no matter how many personal allowances are added to pay to improve retention in Ghana.

**Table 4:** Pay and productivity for different health worker groups and TBAs in Ghana

	***Total mean annual income (USD)***	***% salary out of total income***	***Ratio total pay: GNI/capita [19]***	***Mean hours work/wk***	***Mean no. clients/week***	***Mean no. delivs/wk***	***Pay/client (USD)***	***Pay/delivery (USD)***	***Pay/hour (USD)***
Doctor	14,618	34%	38.5	109.29	257.11	14	1.09	20	2.57
Medical Assistant	5,367	76%	14.1	129.2	172.27	8	0.60	13	0.80
GoG Midwife	5,581	46%	14.7	78.78	119	19	0.90	6	1.36
Private Midwife	2,974	n/a	7.8	111.69	38	4	1.51	14	0.51
Nurse	4,902	58%	12.9	75.7	87.51	14	1.08	7	1.25
Community Nurse	3,606	47%	9.5	56.09	102.5	10	0.68	7	1.24
TBA (trained)	141	n/a	0.4	21.3	8.2	2	0.33	1	0.13
TBAs (untrained)	104	n/a	0.3	19.19	5.87	2	0.34	1	0.10

Comparing pay with outputs, the relatively high number of clients reported by doctors reduces their pay differential, so that the cost per client – $1.09 – is similar to a nurse's (and lower than a private midwife's). MAs though provide the best value of public health workers, at $0.60 per client and $0.80 per hour worked.

## Conclusion

This survey was carried out with health workers and TBAs engaged in deliveries in two regions of Ghana. Its findings shed light on their general working conditions, workload, pay and attitudes, as well as on the changes which have occurred over the past two years and which may be attributable to the delivery fee exemptions scheme which was introduced in this period.

The findings suggest that health workers are fully committed to public services, in terms of their long working hours and lack of private practice. The mean number of clients seen per week by public health workers ranged from 88 for nurses to 257 for doctors. The high number of clients means that doctors cost almost the same as nurses per client seen. This survey does not, however, make any attempt to assess the quality of interaction with clients.

Over the past two years, health workers have seen an increase in workload, accompanied by an increase in pay. Compared with other workers in Ghana doctors are very well paid, largely due to the shortage of doctors throughout the country.

The respondents' views on the exemptions scheme were informed and positive, but also down-to-earth: it has brought an increase in facility deliveries, particularly for the poor, but is threatened by the problem of getting reliable payments from government. For them personally, the effects have been mixed but the increase in workload did not reach the point where it was seriously affecting their morale or ability to cope. The numbers of reported deliveries carried out by the health workers per week (2 for TBAs, 4 for private midwives, 8 for MAs, 10 for CHNs, 14 for doctors and nurses, and 19 for public midwives) were not excessive.

These findings show that a scheme which increases demand for public health services while also sustaining health worker income and morale is workable, if it is well managed, even within the relatively constrained human resources environment of countries like Ghana.

## Competing interests

The author(s) declare that they have no competing interests.

## Authors' contributions

SW developed the research tools, analysed the data and drafted the paper. AK supervised the fieldwork in Volta, analysed the data, and contributed to drafting. MA supervised the field work in CR, and contributed to drafting. All have seen, read and approved the final manuscript.
